# On the Role of Teacher-Student Rapport on English as a Foreign Language Students’ Well-Being

**DOI:** 10.3389/fpsyg.2021.822013

**Published:** 2022-01-21

**Authors:** Sa Li

**Affiliations:** College of Teacher Education, Pingdingshan University, Pingdingshan, China

**Keywords:** teacher-student rapport, well-being, EFL students, academic success, harmonious relationship

## Abstract

Given the centrality of English as a Foreign Language (EFL) students’ wellbeing in their academic success, identifying factors that may be influential in fostering students’ well-being is of high importance. As such, several studies have delved into the role of various personal and interpersonal factors in increasing EFL students’ well-being. However, little attention has been devoted to the function of teacher-student rapport. Besides, no systematic or theoretical review has been conducted in this regard. To address these gaps, the present study intends to illustrate different definitions of student well-being and teacher-student rapport, their sub-components, and their theoretical relations. Building upon the theoretical and empirical bases, the facilitative function of teacher-student rapport in increasing EFL students’ well-being was proved. Some beneficial implications are also discussed.

## Introduction

Relationships with pupils are a key component of any instructional-learning context (Noble et al., 2021). While a negative relationship with students may result in their aggression, apprehension, sadness, anxiety, and stress (Frisby et al., 2014; Alnuzaili and Uddin, 2020), a positive teacher-student relationship may culminate in desirable student-related outcomes (Wubbels et al., 2016; Xie and Derakhshan, 2021). Accordingly, building strong and positive relationships with students has been among the main concerns of all instructors, and EFL (English as a Foreign Language) teachers are not an exception by any means. The positive relationships and connections that teachers aim to create with their pupils is called teacher-student rapport (Catt et al., 2007). Frisby and Martin (2010) defined this construct as an overall feeling between teachers and their students that comprises a mutual and trustworthy bond. Reyes and Von Anthony (2020) further referred to this concept as “a harmonious teacher–student relationship which identified with enjoyment, connection, respect, and mutual trust” (p. 2). To establish such a harmonious relationship, teachers should care about their pupils, pay attention to their efforts, and value their personal comments (Wilson and Ryan, 2013). As put forward by Frisby et al. (2017), being humorous, responsive, and supportive also enables teachers to build a close relationship with their students.

To illustrate the value of teacher-student rapport in classroom contexts, Houser and Hosek (2018) postulated that a positive connection between teachers and students can provide a healthy and friendly atmosphere which is crucial for students’ academic growth and development. In this regard, Culpeper and Kan (2020) also stated that forming strong bonds with pupils not only motivates students to actively engage in different stages of learning, but also empowers them to cope with the challenges and difficulties of the learning process. Xie and Derakhshan (2021) also submitted that positive communication behaviors, including rapport, can lead to favorable student-related outcomes.

Due to the prominence of teacher-student rapport in educational contexts, a large amount of inquiries have delved into the effects of this positive communication behavior on a variety student-related factors, including motivation (e.g., Bouras and Keskes, 2014; Maulana et al., 2014; Koca, 2016; Frisby et al., 2017; Henry and Thorsen, 2018; Zheng et al., 2021), academic engagement (e.g., Lee, 2012; Pianta et al., 2012; Quin, 2017; Roorda et al., 2017; Varga, 2017; Martin and Collie, 2019), academic success (e.g., Estepp and Roberts, 2013; Lammers and Gillaspy, 2013; Glazier, 2016), and academic achievement/learning outcomes (e.g., Yunus et al., 2011; Hughes et al., 2012; Demir et al., 2019; Mellgren, 2020; Wellington, 2021). Nevertheless, the impact of teacher-student rapport on other student-related factors, including well-being, has not been widely examined (Holfve-Sabel, 2014; Graham et al., 2016; Farhah et al., 2021).

The concept of well-being has been generally defined as “the mental health indicator shown by individual ability to cope with pressures in ordinary life, be productive, and be able to contribute to society” (World Health Organization, 2004, as cited in Aulia et al., 2020, p. 2). In Garg and Rastogi’s (2009) words, well-being pertains to “one’s degree of happiness and satisfaction with his/her life, work, and physical and mental health” (p. 43). Building upon Garg and Rastogi’s (2009) definition of well-being, student well-being refers to the amount of satisfaction and happiness that students experience in educational environments (Long et al., 2012). According to Keyes and Annas (2009), student well-being is not only about the presence of happiness and satisfaction or the absence of psychological disorders such as sadness, depression, apprehension, and anxiety. To them, student well-being has also something to do with how students can improve their capabilities to successfully pursue their academic goals. As put forward by Mashford-Scott et al. (2012), students who enjoy an optimum level of well-being can gain higher academic achievements. Similarly, Tian et al. (2015) also noted that students with high level of well-being typically demonstrate a sense of connectedness and attachment to educational environments that lead them toward academic success. Hence, investigating the antecedents or predictors of student well-being seems to be critical. As a response to this necessity, some researchers studied various student-related factors (e.g., Shochet and Smith, 2012; Stallman et al., 2018), teacher-related factors (e.g., Brandseth et al., 2019; Harding et al., 2019; Braun et al., 2020; Lavy and Naama-Ghanayim, 2020), and context-related factors (e.g., Kutsyuruba et al., 2015; Littlecott et al., 2018; Nguyen et al., 2021) that may effectively contribute to higher levels of students’ well-being. Nonetheless, a little attention has been dedicated to teacher-student rapport and its probable effects on student well-being. To put simply, only a few empirical studies (Holfve-Sabel, 2014; Graham et al., 2016) have delved into the impact of teacher-student rapport on students’ level of well-being. Furthermore, no study in a form of review has been conducted to explain the effects of teacher-student rapport on student well-being. To address the aforementioned gaps, the present review inquiry intends to illustrate the effects of teacher-student rapport on EFL students’ well-being by referring to the existing evidence.

### Teacher-Student Rapport

Rapport as an interpersonal behavior pertains to “one’s ability to maintain harmonious relationships based on affinity for others” (Faranda and Clarke, 2004, p. 272). Frisby and Martin (2010) further described this concept as “an overall feeling between two people encompassing a mutual, trusting, and prosocial bond” (p. 147). Extending this definition to the educational context, Lammers and Byrd (2019) conceptualized teacher-student rapport as a mutual bond between teachers and students that inspires them to collaborate with each other in instructional-learning contexts. According to Weimer (2010), respecting students’ ideas, paying attention to their educational needs, and valuing their academic efforts are vital for building a strong rapport with pupils. Similarly, Wilson et al. (2010) argued that those instructors who care about their learners’ needs, interests, and preferences can make a mutual and friendly relationship with them. Further, Estepp and Roberts (2015) also submitted that through verbal (e.g., using humor, asking about learners’ viewpoints, etc.) and non-verbal immediacy cues (e.g., smiling, nodding, etc.) teachers can establish close relationships with their pupils.

### Student Well-Being

The concept of student well-being has been conceptualized differently by several scholars (Graham et al., 2017). To put simply, no consensus has been reached on the definition of student well-being and its’ underlying components (Powell et al., 2018). In their study, De Fraine et al. (2005) defined this construct as “the emotional experience shown by the domination of positive emotion and cognition about the learning environments, instructors, and peers” (p. 299). In another definition, Garg and Rastogi (2009) described student well-being as the extent to which students feel happy and satisfied in educational environments. To characterize the underlying components of student well-being, Miller et al. (2013) divided this construct into three main dimensions, namely *psychological well-being* (i.e., absence of psychological disorders), *school connectedness* (i.e., have a sense of attachment), and *relationships with teachers and classmates* (i.e., healthy relationships with others). In a different categorization, Renshaw et al. (2015) grouped the components of student well-being under four main categories of *sense of connectedness*, *sense of efficacy*, *educational goal*, and *preference of studying*.

According to Brandseth et al. (2019), teachers can remarkably enhance student well-being by supporting their pupils in the process of learning. In this regard, Braun et al. (2020) also postulated that teachers who are able to regulate their negative emotions in classroom contexts can drastically influence students’ well-being in a positive way. It is solely due to the fact that such teachers can easily provide a pleasant learning atmosphere which is highly essential for students’ sense of happiness and satisfaction. As Graham et al. (2016) noted, affective teacher-students relationships can facilitate student well-being as well.

### The Role of Teacher-Student Rapport on English as a Foreign Language Students’ Well-Being

Drawing on the “*rhetorical-relational goal theory*” (Mottet et al., 2006), the impact of teacher-student rapport on EFL students’ well-being can be clearly illustrated. According to Mottet et al. (2006), through various relational and rhetorical communication behaviors such as rapport, language teachers can provide an enjoyable learning atmosphere wherein students will experience a range of positive emotions, including joy, happiness, and contentment, which are directly related to their well-being (Long et al., 2012). Similarly, Maybury (2013) also stated that strong rapport between instructors and learners provide a stress-free atmosphere in which students’ well-being can be dramatically improved. In a similar vein, Luo et al. (2020) also posited that having positive relationships with instructors enables pupils to mitigate their stress, anxiety, and apprehension that are detrimental to their emotional and psychological well-being (He et al., 2018).

## Empirical Studies

Given the fact that teacher-student rapport lies at the heart of education (Xie and Derakhshan, 2021), considerable attention has been devoted to its positive educational outcomes (Roorda et al., 2011; Opdenakker et al., 2012; Lucas-Molina et al., 2015; Ma et al., 2018; Dennie et al., 2019; Meng, 2021; to cite a few). Nonetheless, the positive effects of this communication behavior on students’ well-being have not been widely studied (Holfve-Sabel, 2014; Graham et al., 2016). In her study, Holfve-Sabel (2014) examined the extent to which teacher-student relationship can influence students’ well-being. To do so, some valid measures of the variables were handed out among 1,540 students. The analysis of students’ answers revealed that positive relationships between teachers and students can positively influence students’ well-being in educational environments. In a similar vein, Graham et al. (2016) also probed into the role of teacher-student rapport in primary and secondary students’ well-being. In doing so, 606 students were interviewed. For the sake of triangulation, some reliable questionnaires were also distributed among participants. Analyzing the qualitative and quantitative data, the researchers found a positive and favorable connection between teacher-student rapport and students’ well-being. That is, most of the participants perceived teacher-student rapport as an influential factor in fostering students’ well-being.

## Conclusion and Pedagogical Implications

So far, the existing definitions and classifications of student well-being and teacher-student rapport were fully reviewed. Building upon some related theories (e.g., *rhetorical-relational goal theory*), the positive connection between these two constructs was also illustrated. Additionally, the results of previous inquiries on teacher-student rapport and student well-being were summarized to exemplify the facilitative function of teacher-student rapport in fostering EFL students’ well-being. With regard to the empirical and theoretical evidence, one can safely conclude that close and friendly relationships between EFL teachers and their pupils can greatly contribute to students’ well-being. This finding could be incredibly beneficial for teacher trainers who are directly responsible for the efficient training of instructors. They are expected to teach both pre-service and in-service instructors how to build a strong rapport with pupils. It is largely due to the fact that many instructors, even the experienced ones, do not know how to develop mutual relationships with their students (Hajovsky et al., 2020). The finding of this review study may also be illuminating for EFL/ESL teachers. Due to the undeniable role of teacher-student rapport in enhancing students’ well-being (Maybury, 2013; Luo et al., 2020), teachers are required to attend some workshops or teacher-training courses on teacher-student rapport to become more proficient in establishing positive and close relationships with their pupils. Furthermore, from a positive psychology perspective, it is also suggested that teacher-student rapport can be correlated with such other positive emotions as loving pedagogy, resilience, emotion regulation, grit, engagement, and enjoyment (Wang et al., 2021).

## Author Contributions

The author confirms being the sole contributor of this work and has approved it for publication.

## Conflict of Interest

The author declares that the research was conducted in the absence of any commercial or financial relationships that could be construed as a potential conflict of interest.

## Publisher’s Note

All claims expressed in this article are solely those of the authors and do not necessarily represent those of their affiliated organizations, or those of the publisher, the editors and the reviewers. Any product that may be evaluated in this article, or claim that may be made by its manufacturer, is not guaranteed or endorsed by the publisher.
